# Continuous- and Discrete-Time Stimulus Sequences for High Stimulus Rate Paradigm in Evoked Potential Studies

**DOI:** 10.1155/2013/396034

**Published:** 2013-03-31

**Authors:** Tao Wang, Jiang-hua Huang, Lin Lin, Chang'an A. Zhan

**Affiliations:** School of Biomedical Engineering, Southern Medical University, Guangzhou, Guangdong 510515, China

## Abstract

To obtain reliable transient auditory evoked potentials (AEPs) from EEGs recorded using high stimulus rate (HSR) paradigm, it is critical to design the stimulus sequences of appropriate frequency properties. Traditionally, the individual stimulus events in a stimulus sequence occur only at discrete time points dependent on the sampling frequency of the recording system and the duration of stimulus sequence. This dependency likely causes the implementation of suboptimal stimulus sequences, sacrificing the reliability of resulting AEPs. In this paper, we explicate the use of continuous-time stimulus sequence for HSR paradigm, which is independent of the discrete electroencephalogram (EEG) recording system. We employ simulation studies to examine the applicability of the continuous-time stimulus sequences and the impacts of sampling frequency on AEPs in traditional studies using discrete-time design. Results from these studies show that the continuous-time sequences can offer better frequency properties and improve the reliability of recovered AEPs. Furthermore, we find that the errors in the recovered AEPs depend critically on the sampling frequencies of experimental systems, and their relationship can be fitted using a reciprocal function. As such, our study contributes to the literature by demonstrating the applicability and advantages of continuous-time stimulus sequences for HSR paradigm and by revealing the relationship between the reliability of AEPs and sampling frequencies of the experimental systems when discrete-time stimulus sequences are used in traditional manner for the HSR paradigm.

## 1. Introduction

In studying the auditory evoked potentials (AEPs), high stimulus-rate (HSR) paradigm featuring shorter and irregular interstimulus intervals (ISIs) has been proposed by Delgado and Özdamar [1] and applied to various investigations [2–4]. The specific technique proposed in [1] is generally named as continuous loop average deconvolution (CLAD). For CLAD, the presence of stimulus and silence is, respectively, represented by “1” and “0.” As such a stimulus sweep containing multiple stimulus events is described by a binary sequence. A typical sweep contains a number of “1s” and a large number “0s.” Due to the fact that the ISIs have to be so short for the HSR paradigm that the brain responses to consecutive stimulus events overlap, this binary sequence that constitutes a sweep of stimulus and the sweep response that contains a number of overlapped transient-responses are transformed into the frequency domain to solve the overlapping problem in order to recover the transient responses (see Section 2.1 for more details). This process is usually termed as deconvolution.

As shown by Jewett et al. [5], any chosen stimulus sweep needs to satisfy the noise attenuation property to avoid distorting transient responses in the deconvolution process. As far as this peroperty is concerned, the ISIs must be irregularly distributed in a sweep rather than a fixed ISI as in the conventional recording paradigm. On the other hand, most practical applications require that such ISI-jitters should be as small as possible so that the linear convolution model is valid [5, 6]. The noise attenuation property, which is a major criterion for judging its appropriateness, can be straightforwardly understood in Fourier domain (for details, please see (1d) and (5) below). The generation of stimulus sequences with the desired property is in essence an optimization problem, which is important and dependent on various factors in practice. Typically, a minimal temporal resolution (i.e., the analogy-to-digital (AD) conversion rate for EEG recording) and the number of stimulus events are first chosen for the stimulus sweep for a given experiment. Since the stimulus sequence is optimized at given temporal resolution, we term it as discrete-time sequence. The temporal resolution is crucial for finding a good discrete-time stimulus sequence in that the AD rate is supposed to be as high as possible in order to increase the searching space for a sequence optimization method. However, the use of high temporal resolution imposes challenges for the search of optimal sequence. It either makes the optimization prone to local minima issue or increases computational expense when exhaustive searching strategy is used. Another issue is that when it is chosen, the optimal stimulus sequence should be used at the specific AD rate according to the chosen temporal resolution at which the optimal stimulus sequence is established. 

In reality, different recording systems may not always be operated at the frequency exactly identical to that chosen for stimulus sweep design. When a recording system works at a different frequency, the timing of the onsets of stimulus events in the discrete-time stimulus sweep is to be resampled. It is unclear about the impacts of the resampling and actual AD rate on the deconvolution performance. Moreover, many optimization methods can only deal with continuous variables for the convenience of being exposed to certain mathematic operations [7, 8]. In this case, the optimal timing of stimulus events is a continuous variable, and we term the stimulus sweep as the continuous-time stimulus sequence, which need to be discretized in time domain to be used in actual experiments. The temporal resolution of the discretization is also determined by the AD rate for the actual application. As such, it is important to understand how the AD rate influences the performance of HSR paradigm no matter a resampling or discretization of optimal stimulus sequence is necessary.

To address these critical questions, in this paper, we derived the frequency representation of a continuous-time impulse sequence to solve the deconvolution problem for the HSR paradigm. Using simulated EEGs (based on real AEP data and simulated noises) and four optimized continuous-time stimulus sequences, we demonstrate the applicability and the advantages of continuous-time stimulus sequences for HSR paradigm. We also illustrate the relationship between the AD rate for discretizing a continuous-time sequence and the errors in terms of temporal locations of stimulus impulse, frequency properties of discretized stimulus sequence, and the deconvolved AEP as compared to the ground truth AEP.

## 2. The Convolution Model for HSR Paradigm

### 2.1. Discrete-Time Convolution

Under discrete HSR condition, the observed sweep-response *y*[*n*Δ_*t*_] can be modeled as a circulant discrete convolution between the binary stimulus sequence *s*[*n*Δ_*t*_] and a transient response *x*[*n*Δ_*t*_], that is,

(1a) y[nΔt]=s[nΔt]⊗x[nΔt]+e[nΔt]=∑i=1N(s[nΔt−i]mod⁡N)x[nΔt]+e[nΔt],

where ⊗ denotes circulant convolution; *e*[*n*Δ_*t*_] represents an additive noise term for any undesired contribution to *y*[*n*Δ_*t*_]; Δ_*t*_ is the interval between two discrete samples, or in other word, the reciprocal of the analogue-to-digital (AD) rate *f*_*s*_ (i.e., Δ_*t*_ = 1/*f*_*s*_); *N* is the number of discrete samples for the duration of a stimulus sweep.

Note that the circulant convolution is adopted in (1a). This is because the stimulus sequence *s*[*n*Δ_*t*_] is delivered to the subject repetitively in the HSR paradigm. As such, *y*[*n*Δ_*t*_], *x*[*n*Δ_*t*_], and *e*[*n*Δ_*t*_] are of the same length *N*, or the same time duration *T* = *N*/*f*_*s*_. In practice, the recorded raw electroencephalograms (EEGs) are epoched according to the stimulus sweep rather than to individual stimulus impulse (each stimulus sweep contains a number of stimulus impulses depending on the particular experiment design), and averaged over a number of sweeps to attenuate the noise level. Equation (1a) is usually referred to the case after averaging.

Equation (1a) can be represented in Fourier domain as

(1b) $$\begin{array}{c} Y\left[jmf_{0}\right]=S\left[jmf_{0}\right]X\left[jmf_{0}\right]+E\left[jmf_{0}\right], \end{array}$$

where *f*_0_ = 1/*T* and the capital letters correspond to the discrete Fourier transforms of their counterparts (e.g., *X*[*jmf*_0_] denotes the discrete Fourier transform of *x*[*n*Δ_*t*_]),

(1c) X[jmf0]=∑i=1Nx[nΔt]+e−j(2π/N)mn,   m=1,2,…,N.

From (1b) one can solve the transient response *x*[*n*Δ_*t*_] in the frequency domain (i.e., *X*[*jmf*_0_]) in a straightforward way using an inverse filter as done by the CLAD [1]. Consider

(1d) X^[jmf0]=Y[jmf0]S[jmf0]+E[jmf0]S[jmf0]=X[jmf0]+E[jmf0]S∗[jmf0]|S[jmf0]|.

Based on (1d), it is obvious that distortion of the solution *X*[*jmf*_0_] can happen at some frequency bins where |*S*[*jmf*_0_]| approaches to zeros, since small |*S*[*jmf*_0_]| can amplify the noise components *E*[*jmf*_0_]. 

To address this issue, Jewett et al. [5] propose a scheme to examine the values of |*S*[*jmf*_0_]| within the frequency band of interest and make sure that these values are larger than a preset threshold. In a following study, Jewett et al. [7] offered a number of binary stimulus sequences according to this criterion.

### 2.2. Continuous-Time Convolution

Knowing the frequency property of a stimulus sequence can assist the assessment of the noise attenuation performance of the inverse filter for the deconvolution problem [8]. Early studies such as those by Jewett et al. [5, 7] guiding the selection of appropriate stimulus sequences have made the HSR scheme practical for actual applications and thus significantly contributed to the development of HSR paradigms. However, the optimal stimulus sequences used in the literature [7] so far are AD rate dependent and thus not generalizable due to the need of resampling when the actual AD rate differs from that used for stimulus sequence optimization. As such, there are two major drawbacks in optimizing the stimulus sequences in discrete form. First, the optimized sequence may not be optimal if the sequence is resampled with a different rate. This means that it is impossible to generate a sequence for general use; instead, optimization algorithm is to be employed to generate a good sequence according to the AD rate for each given experiment. Second, optimization in discrete form makes it hard to know how the AD rates influence the performance of an optimized sequence. 

In this section, we derive the continuous-time convolution relationship between the stimulus sequence and transient response, and the estimation of transient response in a general form. 

Similar to the *y*[*n*Δ_*t*_] in (1a), the observable EEG sweep *y*(*t*) can be represented as the circulant convolution between transient response *x*(*t*) and stimulus *s*(*t*), with error term *e*(*t*):

(2) y(t)=s(t)⊗x(t)+e(t)=∫0Ts(τ)x(t−τ)mod⁡Tdτ+e(t), t∈[0,T].

Since all the variables (except the error term *e*(*t*)) in (2) can be considered as periodical functions (with a period of *T*) due to the repetitive stimulation manner, the key difference here from (1a) is that the stimulus events in *s*(*t*) can happen at any time point *t* within the period *T*, rather than the discrete time points determined by the AD rate. The Fourier transform of such signals as *x*(*t*) is in discrete form:

(3) $$\begin{array}{c} F\left[x\left(t\right)\right]=X\left(jkf_{0}\right)=\int_{0}^{T}x\left(t\right)e^{-j2\pi kf_{0}}dt, \end{array}$$

where *F*[·] denotes Fourier transform operator, and *f*_0_ = 1/*T* represents the repetition rate of the stimulus sweep and thus the discrete frequency resolution for signals (e.g., *X* in (3)) to be presented in Fourier domain. We can rewrite (2) in Fourier domain:

(4) $$\begin{array}{c} Y\left(jkf_{0}\right)=S\left(jkf_{0}\right)X\left(jkf_{0}\right)+E\left(jkf_{0}\right). \end{array}$$

The transient response *x*(*t*) can be estimated in Fourier domain as

(5) X^(jkf0)=Y(jkf0)S(jkf0)+E(jkf0)S(jkf0)=X(jkf0)+E(jkf0)S∗(jkf0)|S(jkf0)|.

Distortion of the estimated transient response is introduced in the second term of the right hand side of (5). The noise term *E*(*jkf*_0_) is to be amplified by the inverse filter *S*(*jkf*_0_)^−1^ at some frequency bins where |*S*(*jkf*_0_)^−1^| is small. As such, it is critical to study in detail the properties of the inverse filter *S*(*jkf*_0_)^−1^ and we will do so in Section 2.3. 

Equation (5) shows that as far as these continuous periodical signals are concerned, the error term's contribution to the estimation of *x*(*t*) depends only on *f*_0_ but not the AD rate *f*_*s*_ as in (1d). Note that the frequency range of the solution can be infinite in theory, but in practice, the energy of transient signal *x*(*t*) is bounded within a relatively narrow frequency band of interest and only the continuous-time stimulus sequences can be of unlimited frequency range, which is to be detailed below. 

### 2.3. The Properties of Continuous-Time Stimulus Sequence

A stimulus sequence *s*(*t*) for one experiment sweep can be described as a *P* impulses train. We use delta functions of delay *t*_*p*_(*p* = 1,2,…, *P*) to represent the occurrence of stimulus impulses and their summation to represent the stimulus sweep:

(6) $$\begin{array}{c} s\left(t\right)=\sum_{p=1}^{P}\delta \left(t-t_{p}\right). \end{array}$$

**Figure 1:**
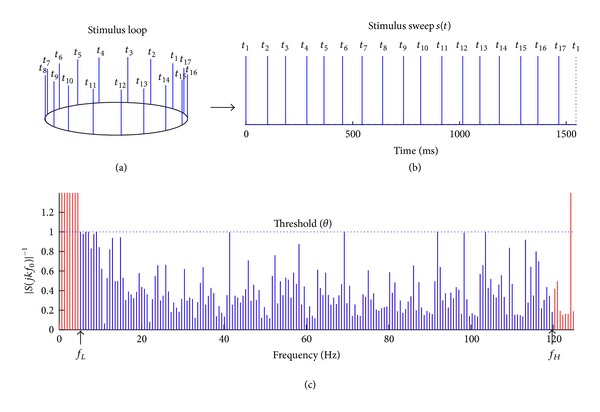
(a) A schematic diagram of a stimulus loop that contains 17 individual stimulus impulses to indicate the repetitive presentations in an experiment. (b) A sweep of stimulus sequence with impulses occurring at *t*_*p*_(*p* = 1,2, 3,…, 17) to represent a period of stimulus loop in time domain (*T* = 1545.5 ms; *f*_0_ = 0.647 Hz). (c) A portion of the Fourier spectrum of the inverse filter |*S*_*T*_(*jkf*_0_)|^−1^. Note that within the frequency band of interest ([*f*_*L*_′, *f*_*H*_′] = [5, 120] Hz), |*S*_*T*_(*jkf*_0_)|^−1^ is all below the threshold (*θ* = 1).

In this continuous-time form of stimulus sequence, the *t*_*p*_ is a continuous variable, that is, *t*_*p*_ ∈ [0, *T*].  Figure 1(a) shows an example sweep with stimulus impulses occurring at 17 time points. The Fourier transform of (6) is

(7) $$\begin{array}{c} F\left[s\left(t\right)\right]=S\left(jf\right)=\sum_{p=1}^{P}e^{-j2\pi {ft}_{p}}. \end{array}$$

This is a continuous function in the frequency domain [9]. In a given experiment, the stimulus sweep (Figure 1(a)) is repetitively delivered to the subject to stimulate the brain responses for EEG recording. As such, the whole stimulus sequence (Figure 1(b)) is modeled as a convolution between the impulses train *s*(*t*) and a summation of delta functions *δ*_*T*_(*t*):

(8) $$\begin{array}{c} s_{T}\left(t\right)=s\left(t\right)*\delta_{T}\left(t\right), \end{array}$$

where *δ*_*T*_(*t*) is a periodic function with period *T* defined as

(9) $$\begin{array}{c} \delta_{T}\left(t\right)=\sum_{q=-\infty }^{+\infty }\delta \left(t-qT\right). \end{array}$$

The Fourier transform of (9) is

(10) $$\begin{array}{c} F\left[\delta_{T}\left(t\right)\right]=2\pi f_{0}\sum_{k=-\infty }^{\infty }\delta \left(f-kf_{0}\right). \end{array}$$

Equation (10) shows that the Fourier transform of a periodical delta sequence is also a delta train with the interval of *f*_0_. 

Based on (8) and (10), the Fourier transform of the periodical stimulus *s*_*T*_(*t*) is

(11) $$\begin{array}{c} S_{T}\left(jf\right)=2\pi f_{0}S\left(jf\right)\sum_{k=-\infty }^{+\infty }\delta \left(f-kf_{0}\right). \end{array}$$

Equation (11) indicates that the spectrum *S*_*T*_(*jf*) is a discrete sampling of the continuous function *S*(*jf*) in (7) by the delta function train at the interval of *f*_0_. Plugging *S*(*jf*) in (7) into (11), we get

(12) $$\begin{array}{c} S_{T}\left(jf\right)=2\pi f_{0}\sum_{p=1}^{P}\;\sum_{k=-\infty }^{+\infty }e^{-j2\pi {ft}_{p}}\delta \left(f-kf_{0}\right), \end{array}$$

which can be rewritten as follow given the sampling property of the delta function *δ*(*f* − *kf*_0_):

(13) $$\begin{array}{c} S_{T}\left(jkf_{0}\right)=2\pi f_{0}\sum_{p=1}^{P}e^{-j2\pi f_{0}t_{p}}. \end{array}$$

Equation (13) is the Fourier spectrum of the stimulus impulse-sequence, which includes infinite number of frequencies defined by *kf*_0_ since *k* can be any integer. In real experiment, however, signals and noises are limited within a frequency band, say [*f*_*L*_, *f*_*H*_]. While *f*_*L*_ and *f*_*H*_ can be of arbitrary real values in theory, we should round off them to the multiples of *f*_0_ in practice, say [*f*_*L*_′, *f*_*H*_′], in the discrete frequency domain:

(14) $$\begin{array}{c} \left[f_{L}^{\prime },f_{H}^{\prime }\right]=\left[k_{L}f_{0},k_{H}f_{0}\right], \end{array}$$

where *k*_*L*_ = ⌈*f*_*L*_/*f*_0_⌉ and *k*_*H*_ = ⌊*f*_*H*_/*f*_0_⌋ are the frequency domain indexes for the frequency band of interest. 

As shown in (5), the inverse filter *S*(*jkf*_0_)^−1^ critically determines the error term's contribution to the distortion of deconvolved transient response *x*(*t*). To limit the distortion, it is necessary to constrain the Fourier energy distribution of the stimulus sequence as follows so that the noise term *E*(*jkf*_0_) in (5) is at least not amplified. Assume

(15) $$\begin{array}{c} {\left|S_{T}\left(jkf_{0}\right)\right|}^{-1}\leq \theta ,\;k=k_{L},\ldots ,k_{H}, \end{array}$$

where *θ* is a threshold usually set at 1 to make sure that the noise term within the frequency bins of interest is at most maintaining its original energy if not attenuated by the inverse filter. 

Figure 1(c) illustrates that the example stimulus loop in Figure 1(a) meets this criterion in that the inverse filter satisfies |*S*_*T*_(*jkf*_0_)|^−1^ < 1 in the chosen frequency band [*f*_*L*_′, *f*_*H*_′].

To further evaluate the overall quality of the stimulus sequence in (6), a measure called noise gain factor (NGF) can be defined accordingly [10] as

(16) $$\begin{array}{c} \text{NGF}=\frac{1}{f_{H}^{\prime }-f_{L}^{\prime }}\sum_{k=k_{L}}^{k_{H}}{\left|S_{T}\left(jkf_{0}\right)\right|}^{-1}, \end{array}$$

which represents the average of noise gain factor at each frequency *kf*_0_.

## 3. Experiments and Results 

### 3.1. Stimulus Impulse Sequences

In this section, we generate continuous-time stimulus sequences to be used for examining the impact of AD rates on the performance of inverse filtering in solving the transient AEPs. Using various optimization methods [11], these stimulus sequences can be found to satisfy the constraint in (15). Here we employed a modified optimization method called differential evolution algorithm [12] to obtain the optimal continuous-time stimulus sequences. The value of threshold *θ* in (15) was set to 1. Since the details of the optimization are beyond the scope of this paper, we directly provide the four impulse sequences (Table 1) generated for our study. 

These four sequences are given in the form of ISI-series which can be expressed as Δ*t*_*p*_ = *t*_*p*+1_ − *t*_*p*_, where *t*_*p*_ = *T* if *p* = *P*. Note that the temporal resolution of the stimulus events in the sequence is infinity in theory. In Table 1, we show Δ*t*_*p*_ in the second decimal place only. These sequences are used to study the characteristics of 40 Hz steady-state responses which is a main application of HSR paradigms [2, 4]. Here, the jitter ratio (JR) in Table 1 is defined as JR = [max⁡{Δ*t*_*p*_} − min⁡{Δ*t*_*p*_}]/max⁡{Δ*t*_*p*_} in percentage to measure the inhomogeneity of the stimulus interval. The applications of HSR paradigms usually requires a low jitter (small JR) stimulation to approach the case of steady state recordings while satisfying the constraint of (15) within the frequency band of interest (8–122 Hz, see Figures 4 and 5) in which the majority of energy of the simulated EEG signal falls.

### 3.2. EEG Data Simulation

In actual experiments, the recorded EEGs (including transient AEPs and noises) are band-limited signals. They are digitized in time and amplitude according to Nyquist-Shannon sampling theorem. In this study, we use a real AEP signal previously measured using CLAD method [8] as the AEP component (*x*(*t*)) and the additive noise (i.e., *e*(*t*)) generated from a 1/*f* process [13] to simulate background EEGs mixed with inherent artifacts and noise. In our practice, the *e*(*t*) is filtered by a band-pass filter to eliminate frequency outside [8, 500] Hz as a recording system usually does in experiments for the recording of middle latency response and 40 Hz steady state response. Note that *x*(*t*) and *e*(*t*) are both band-limited signals which in this case fall in frequency range [8, 500] Hz. In theory, there is no error when resampling them at a different AD rate as long as the sampling theorem is satisfied. In this study the original signals *x*(*t*) and *e*(*t*) were obtained at AD rate of 20 kHz and then resampled into other rates as needed. We chose a frequency band [8, 500] Hz for the simulated EEG signal to emulate the actual signal from recorded EEGs, which usually have a cut off frequency at 500 Hz in real experiment. However, this frequency band is broader than that ([8, 122] Hz) we used in optimizing our stimulus sequence in Section 3.1. Theoretically, we should avoid this inconsistency by either generating stimulus sequence satisfactory within [8, 500] Hz or filtering the EEG signal to [8, 122]Hz. In practice, we found it was much more difficult, if not impossible, to optimize the stimulus sequence for broad frequency range. Although the stimulus sequences in this paper are optimized for a frequency band narrower than that for the EEG signal, there was no data point of |*S*_*T*_(*jkf*_0_)|^−1^ measure within the range of [0,  500]Hz to be extremely large. Moreover, the energy of EEG signals beyond range [8, 122] Hz is very low since the AEP is a very narrow band signal and the noise is simulated using a 1/*f* process. As such, noise will not be over amplified by the inverse filtering in this study and their contribution to the distortion of deconvolved AEP is limited. 

### 3.3. Errors Introduced by the Temporal Discretization

The interstimulus interval of continuous-time stimulus sequence can be discretized at required temporal resolution. The discretization will introduce round off errors no matter how high is sampling frequency *f*_*s*_. Accordingly, the Fourier of the discretized counterpart will differ from that of the original continuous-time stimulus sequence. 

The discretized temporal location *t*_*p*_′ of a stimulus impulse at *t*_*p*_(*p* = 1,2, …, *P*) with respect to the onset of a stimulus-sweep is

(17) $$\begin{array}{c} t_{p}^{\prime }=\text{round}\frac{\left(t_{p}f_{s}\right)}{f_{s}}. \end{array}$$

The normalized root-mean-square error is defined as an overall measure of the errors caused by the temporal discretization of the continuous-time stimulus impulse sequence

(18) $$\begin{array}{c} \gamma_{t}=\frac{P}{T}\sqrt{\frac{1}{P}\sum_{p=1}^{P}{\left(t_{p}-t_{p}^{\prime }\right)}^{2}}. \end{array}$$

**Figure 2:**
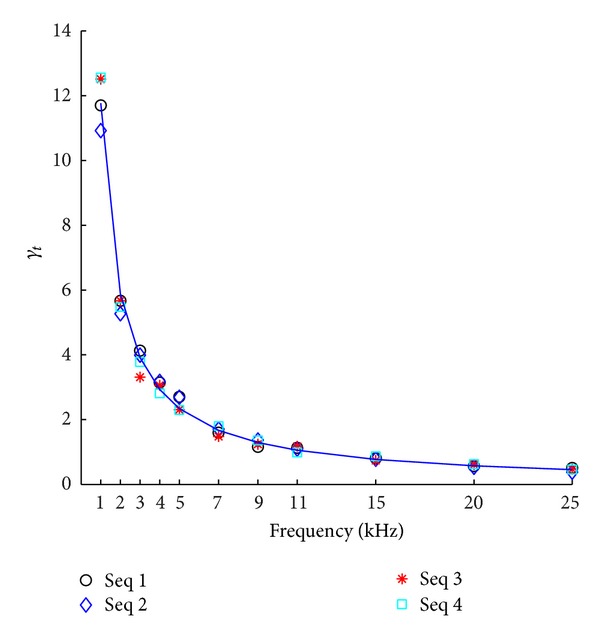
The graphic representation of the relationship between *γ*_*t*_ and *f*_*s*_. The data points for four datasets are fit using a reciprocal function: *γ*_*t*_ = 11.78/*f*_*s*_ − 0.01363, (*R* ^2^ = 0.9982). The location of maximal curvature is at *f*_*s*_ = 3.43 kHz.

To examine the error introduced by the temporal discretization of four stimulus sequences in Table 1, we calculated *γ*_*t*_ at different temporal resolutions determined by the AD rates (from 1 kHz to 25 kHz). The results are presented in Figure 2. Consistent with results for the four sets of stimulus sequences, the *γ*_*t*_ decreases as sampling frequency increases. The results can be fit by a reciprocal function: *γ*_*t*_ = 11.78/*f*_*s*_ − 0.01363, (*R* ^2^ = 0.9982) and the location of maximal curvature *f*_*s*_ = 3.43 kHz.

As seen above, the temporal discretization introduces errors of the temporal location of the stimulus impulses, causing the difference between *S*_*T*_(*jkf*_0_) and *S*[*jkf*_0_]. We need to examine whether an optimized continuous-time stimulus sequence *s*(*t*) still satisfies the constraint in (15) after discretization. We define relative root-mean-square error in frequency domain as

(19) $$\begin{array}{c} \gamma_{f}=\frac{\sqrt{\sum_{k=k_{L}}^{k_{H}}{\left(\left|S_{T}\left(jkf_{0}\right)\right|-\left|S[jkf_{0}]\right|\right)}^{2}}}{\sqrt{\sum_{k=k_{L}}^{k_{H}}{\left|S\left(jkf_{0}\right)\right|}^{2}}}\times 100\%. \end{array}$$

**Figure 3:**
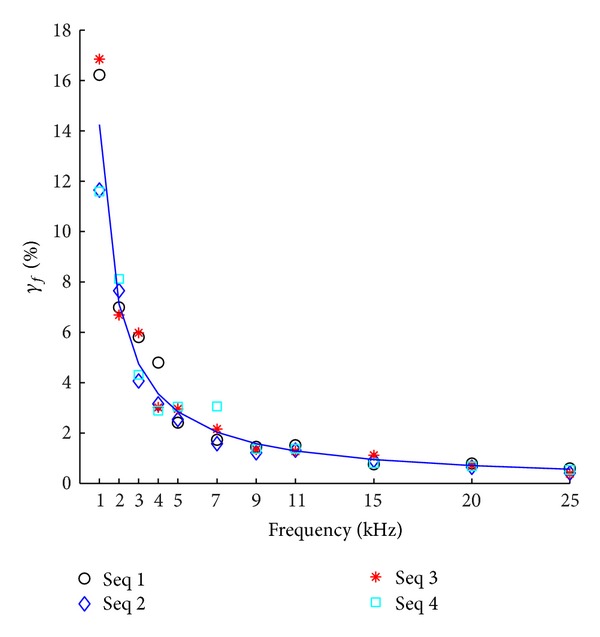
The graphic representation of the relationship between *γ*_*f*_ and *f*_*s*_. The data points for four datasets are fit using a reciprocal function: *γ*_*f*_ = 14.25/*f*_*s*_ − 0.003574, (*R* ^2^ = 0.9983). The location of maximal curvature is at *f*_*s*_ = 3.77 kHz.

Using the same four dataset in Table 1, we calculate *γ*_*f*_ with respect to each AD rate from 1 kHz to 25 kHz. The resulting *γ*_*f*_ is shown in Figure 3. Again, the data points can be well-fit with a reciprocal function: *γ*_*f*_ = 14.25/*f*_*s*_ − 0.003574, (*R* ^2^ = 0.9983), and the location of its maximal curvature is at *f*_*s*_ = 3.77 kHz.

Figure 3 shows that the error of stimulus sequence in Fourier domain caused by the temporal discretization decreases with the increase of sampling rate. But the error will not completely disappear. Based on the fit function *γ*_*f*_ = 14.25/*f*_*s*_ − 0.003574, the maximal curvature occurs at *f*_*sc*_ = 3.77 kHz, indicating that, in the range [0, 3.77] kHz, *γ*_*f*_ decreases rapidly with the increase of *f*_*s*_. However, *γ*_*f*_ decreases at a much low rate when *f*_*s*_ is greater than 3.77 kHz. These results suggest that the *γ*_*f*_ is very sensitive to low AD rate, and that it is critical to increase the AD rate to a frequency higher than 3.77 kHz.

**Figure 4:**
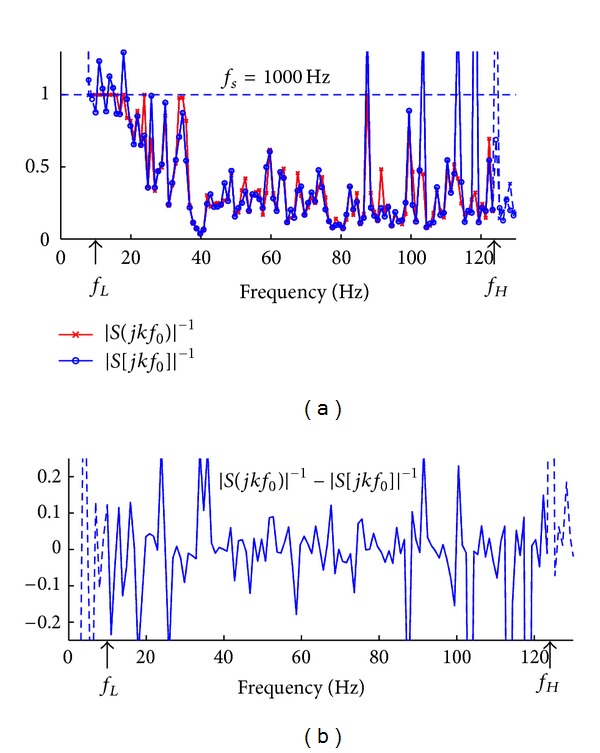
Comparison with respect to the |*S*_*T*_(*jkf*_0_)|^−1^ measure between the continuous-time stimulus sequence and its discrete-time counterpart at discretization frequency of *f*_*s*_ = 1 kHz. (a) The plot of the |*S*_*T*_(*jkf*_0_)|^−1^ measure for the continuous-time stimulus sequence (data points in “*x*”) and that for the corresponding discrete-time stimulus sequence (data points in “*o*,” discretization frequency *f*_*s*_ = 1 kHz). (b) The difference between the |*S*_*T*_(*jkf*_0_)|^−1^ measures, respectively, for continuous-time and discrete-time stimulus sequences.

Here we use one stimulus sequence to exemplify the differences caused by discretization at two different AD rates: *f*_*s*1_ = 1 kHz, which is below the critical *f*_*sc*_ (3.77 kHz) and *f*_*s*2_ = 20 kHz, which is above the critical *f*_*sc*_ (3.77 kHz).  Figure 4(a) illustrates the |*S*_*T*_(*jkf*_0_)|^−1^ measure of the continuous-time stimulus sequence (data points labeled with cross “*x*”) and that of the corresponding discrete-time stimulus sequence (data points labeled with open circle “*o*”) at an AD rate of *f*_*s*_ = 1 kHz. Within the frequency range of interest [*f*_*L*_ = 8 Hz, *f*_*H*_ = 122 Hz], the inverse filter based on the discretized stimulus sequence satisfies (15) at most frequency bins. However, at some frequency bins, its |*S*_*T*_(*jkf*_0_)|^−1^ measure is greater than 1. The difference between the continuous-time and discrete-time stimulus sequence at each frequency bin is plotted in Figure 4(b). Clearly, the discretization causes errors at most frequency bins at amplitudes within the range from −0.25 to 0.25. 

**Figure 5:**
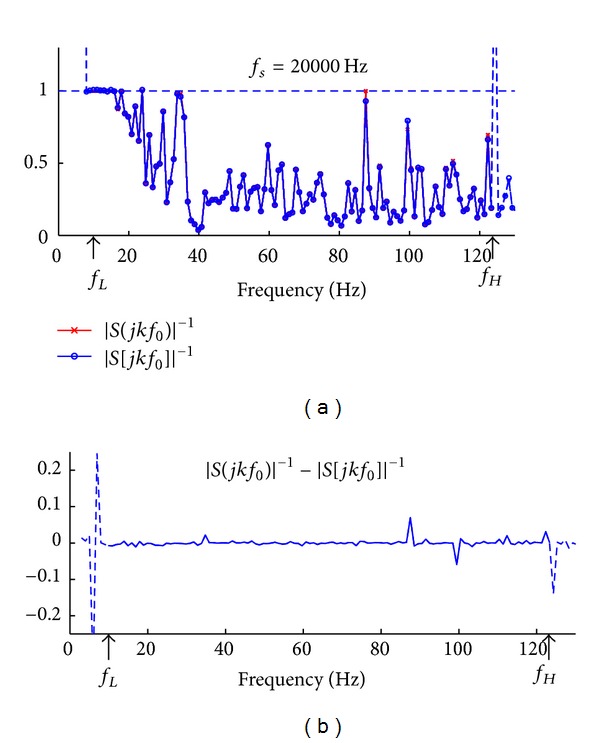
Comparison with respect to the |*S*_*T*_(*jkf*_0_)|^−1^ measure between the continuous-time stimulus sequence and its discrete-time counterpart at discretization frequency of *f*_*s*_ = 20 kHz. (a) The plot of the |*S*_*T*_(*jkf*_0_)|^−1^ measure for the continuous-time stimulus sequence (data points in “*x*”) and that for the corresponding discrete-time stimulus sequence (data points in “*o*”, discretization frequency *f*_*s*_ = 20 kHz). (b) The difference between the |*S*_*T*_(*jkf*_0_)|^−1^ measures, respectively, for continuous-time and discrete-time stimulus sequences.

Figure 5 shows the similar contents as Figure 4, except that the continuous-time stimulus sequence is discretized at a higher AD rate of *f*_*s*_ = 20 kHz.  Figure 5(a) depicts the |*S*_*T*_(*jkf*_0_)|^−1^ measures for continuous-time (data points labeled with “*x*”) and discrete-time (data points in open circles “*o*”) stimulus sequences. Again, the |*S*_*T*_(*jkf*_0_)|^−1^ measure within most frequency bins for the discrete-time stimulus sequence satisfies (15). However, we can also see some data points stick out of the threshold of 1. The difference between the |*S*_*T*_(*jkf*_0_)|^−1^ measures for the continuous-time and discrete-time stimulus sequences is shown in Figure 5(b). We can see that at *f*_*s*_ = 20 kHz, the difference at most frequency bins is close to zero. When compared to Figure 4(b), Figure 5(b) clearly show that *f*_*s*_ = 20 kHz has substantial advantage over *f*_*s*_ = 1 kHz in maintaining the discrete stimulus sequence satisfactory to the condition described in (15). Importantly, if the AD rate is low, the discrete stimulus sequence may violate the condition and cause overamplification of noise components in the recorded EEG and distort the deconvolved transient AEPs. The results also suggest that using AD rate much higher than the Nyquist sampling rate is necessary to minimize the error introduced from temporal discretization.

### 3.4. Noise Attenuation Effects of Discretization Frequency

Although the continuous-time stimulus sequence *s*(*t*) is frequency unlimited (*S*(*jkf*_0_) in Fourier domain, where *k* can be any integer), the frequency range of interest is determined by the nonzero values of the product of *S*(*jkf*_0_)and *X*(*jkf*_0_) as indicated in (4). In this study, the recorded EEG is band-pass filtered within [8–500] Hz. The transient response *x*(*t*) is obtained by applying inverse Fourier transform on $\hat{X}(jkf_{0})$ in (5):

(20) x^c(nΔt)=F−1{X^(jkf0)}, n=1,2,…,N;     8 Hz  ≤kf0≤500 Hz.

Likewise, the inverse Fourier transform of $\hat{X}\left[jmf_{0}\right]$ in (1d) will give the time domain signal for the discrete binary sequence:

(21) x^d[nΔt]=F−1{X^[jmf0]}, n=1,2,…,N;     8 Hz  ≤mf0≤500 Hz.

To quantify the difference between the estimated and the true solutions for both continuous and discrete stimulus sequence, we can define root mean square error for ${\hat{x}}_{c}(n\Delta_{t})$

(22) $$\begin{array}{c} \gamma_{x_{c}}=\frac{\sqrt{\sum_{n=1}^{N}{\left({\hat{x}}_{c}\left[n\right]-x\left(n\Delta_{t}\right)\right)}^{2}}}{\sqrt{\sum_{n=1}^{N}{x\left(n\Delta_{t}\right)}^{2}}}\times 100\%. \end{array}$$

By replacing ${\hat{x}}_{c}(n\Delta_{t})$ with ${\hat{x}}_{d}\left[n\Delta_{t}\right]$ in (22), we can define the root mean square error *γ*_*x*_*d*__ for ${\hat{x}}_{d}(n\Delta_{t})$. As stated previously, an original copy of additive 1/*f*random noise is then rescaled and/or resampled to accommodate conditions with different AD rates and signal-to-noise ratios (SNRs), where the latter is defined as

(23) $$\begin{array}{c} \text{SNR}=20\log\frac{\sqrt{\sum_{n=1}^{N}{x\left(n\Delta_{t}\right)}^{2}}}{\sqrt{\sum_{n=1}^{N}{e\left(n\Delta_{t}\right)}^{2}}}. \end{array}$$

**Figure 6:**
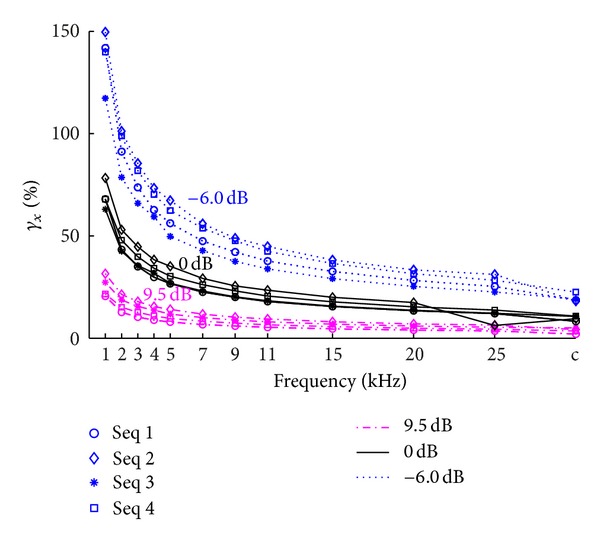
The errors between estimated and true solution at various AD rates (1–25 kHz) as well as the case of continuous-time sequence (tick “c” in the horizontal coordinate). Four sequences are examined under three SNR conditions (see the legend). *γ*_*x*_ in the vertical coordinate implies both errors of *γ*_*x*_*c*__ and *γ*_*x*_*d*__.

To examine the relationship between AD rate and *γ*_*x*_*c*__, we simulated the discretization at *f*_*s*_ = 1–25 kHz and three SNR conditions (9.5 dB, 0 dB, and −6.0 dB). The resulting *γ*_*x*_*c*__ is shown in Table 2, where in the first column (AD rate = Inf) means the errors for the continuous-time stimulus sequence condition and *γ*_*x*_*c*__ results for this condition serve the references for all other AD rates. From Table 2, we can see that the errors for high SNR EEGs are much smaller than those for low SNR EEGs, and the errors decrease as AD rate increases. These phenomena are consistent across four stimulus sequences.  Figure 6 is a graphic representation of the results. It shows that low SNR EEGs are more vulnerable to low AD rates, and the increase AD rate is more beneficial for low SNR signal on the other hand.

**Figure 7:**
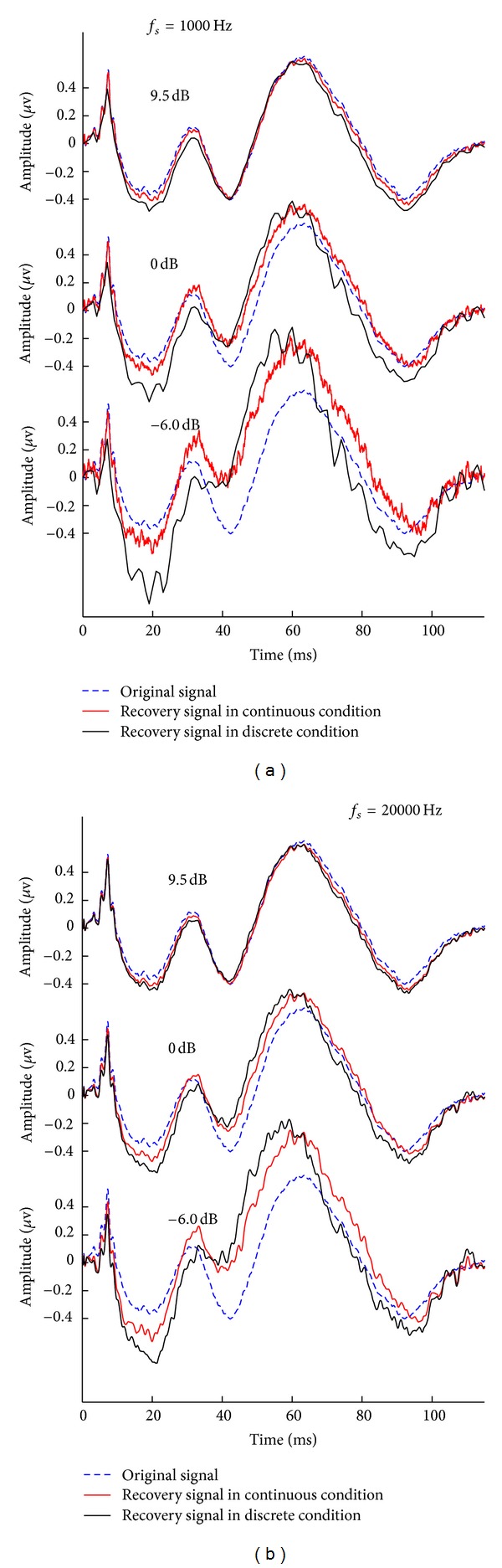
Comparison of transient AEPs solved by CLAD method at different SNR conditions and at two representative discretization frequencies. (a) AEPs (the original in dashed blue, the one recovered based on continuous-time stimulus sequence in thin red, and the one recovered based on discrete-time stimulus sequence in black). The discretization frequency (AD rate) *f*_*s*_ = 1 kHz; and the SNRs for three panels from top to bottom are, respectively, 9.5, 0 and −6.0 dB. (b) Same as (a) except the discretization frequency *f*_*s*_ = 20 kHz.

To gain more straightforward understanding of above results, we choose to present the resulting AEPs from different SNR conditions and at two representative AD rates.  Figure 7(a) shows the results at AD rate of 1 kHz. It is clear that the AEPs recovered from high SNR EEGs (the top panel) using continuous-time and discrete-time stimulus sequence are both very close to the original AEP used for the simulation study. However, the difference between the recovered AEPs becomes significant when the SNR is low (the third panel in Figure 7(a)). At same time, we can see that the AEPs recovered using continuous-time stimulus sequence are closer than the ones from discrete-time stimulus sequence to the original AEP.  Figure 7(b) shows the results at AD rate of 20 kHz. Similar to the phenomena in Figure 7(a), the AEPs (the top panel in Figure 7(b)) recovered from high SNR EEGs are very close to the original AEP used in this study. When the SNR is getting worse, for example −6 dB as in the third panel in Figure 7(b), the recovered AEP is less close to the original AEP. Consistent to that exemplified in Figure 7(a), the AEPs recovered based on continuous-time stimulus sequence better resemble the original AEP than those based on discrete-time stimulus sequence. Moreover, the AEPs from high AD rate (*f*_*s*_ = 20 kHz) of discretization resemble more closely to the original AEP than those from low AD rate of discretization. As such, it becomes obvious that increasing AD rate will reduce the distortions in the recovered transient AEP. More importantly, this positive impact of high AD rate is more significant for low SNR recordings.

## 4. Discussion and Conclusion

Solving the transient AEPs in frequency domain under HSR paradigm has been investigated and implemented recently using discrete-time stimulus sequences (e.g., in [1–5, 7, 8, 10]). Since the frequency characteristics of a stimulus sequence critically influence the performance of deconvolution algorithms in the presence of noises in EEG recordings, one needs to select or generate appropriate sequences to satisfy certain criteria (e.g., (15)). However, it is challenging to generate an optimal discrete-time stimulus sequence when the occurring time points of stimulus impulses are subject to the constraint of the AD rate of the experiment systems. This is because theoretically it only has a limited number of possible solutions. Although continuous sequences generated by an optimum method might be ready for use, one needs to answer two more questions: (1) how to present such sequences into the practical recordings in digital form; (2) how to quantify the errors and impacts due to the discretization of the continuous-time stimulus sequences. 

This paper answers the first question by explicating the frequency presentation of continuous-time stimulus sequences and its use in solving the transient AEP using deconvolution algorithm. As detailed before, the repetitive continuous-time stimulus sequences have discrete frequencies in the Fourier domain and as such the convolution model and deconvolution algorithm can be similarly presented as for the discrete-time time system. In practice, the continuous-time stimulus sequences can be approximated using very high temporal resolution discrete-time stimulus sequences. For example, the temporal resolution for the stimulus sequences in Table 1 can be rounded to the 4th decimal place, equivalent to a sampling frequency at MHz level, which is well above the characteristic frequency (~4 kHz) in Figures 2 and 3 and makes the discretization errors negligible in applications. Furthermore, simulation studies are used to examine the applicability of the theory and quantify the errors and impacts when a continuous-time stimulus sequence is temporally discretized. The results show that using continuous-time stimulus sequences in the HSR paradigm has significant advantages over using discrete-time stimulus sequences in recovering the transient AEPs. This study also reveals a reciprocal relationship between errors introduced by the discretization of continuous-time stimulus sequence into discrete-time counterpart and the discretization frequencies. Note that we here used the absolute errors to quantify the impact of sampling rate of discrete-time stimulus sequences. But absolute errors should not be considered the only choice. In fact, other metrics such as correlation coefficient can be a good measure when the shape of waveform is of the researchers' only concern. In typical AEP studies, researchers are usually interested in both the shape and amplitude of the signals. In any case, the results in this paper suggest that when discrete-time stimulus sequence is used, a high frequency system is more likely to offer reliable recovered AEPs under HSR paradigm. 

To conclude, this study demonstrates the applicability and advantages of continuous-time stimulus sequences for AEP studies using HSR paradigm and reveals the reciprocal relationship between the errors in recovered AEPs and the sampling frequencies of the experimental systems when discrete-time stimulus sequences are used in traditional manner for the HSR paradigm.

## Figures and Tables

**Table 1:** Four optimized stimulus impulse sequences obtained using differential evolution algorithm.

Sequence (ID, JR, NGF)	Stimulus interval (Δ*t*_*p*_ in ms, rounded to the second decimal place for this table only)
Seq 1 11.95%, 0.43	27.21, 22.34, 27.16, 21.40, 21.43, 23.26, 27.18, 24.38, 26.19, 21.48, 27.16, 24.32, 27.05, 23.57, 24.02, 26.30, 27.04, 21.40, 23.41, 27.21, 22.16, 24.62, 21.47, 22.37, 22.54, 27.19, 27.21, 21.40, 21.40, 21.49, 27.21, 22.57, 25.52, 25.15, 27.21, 21.40, 21.40, 27.21, 24.92, 21.40, 25.93, 27.21, 27.21, 21.43, 21.74, 22.23, 27.20, 26.74, 27.21, 21.40, 24.39, 21.49, 24.38, 27.21, 22.54, 27.20, 21.40, 21.86, 27.21, 21.97, 22.59, 25.09, 27.15, 21.48, 26.27

Seq 2 12.40% 0.50	23.49, 21.91, 27.96, 27.96, 21.79, 21.79, 24.88, 27.96, 24.36, 21.79, 26.27, 23.13, 26.82, 27.96, 21.79, 27.96, 23.67, 22.83, 27.96, 21.79, 22.40, 27.96, 27.96, 21.91, 21.79, 27.96, 23.33, 27.96, 21.79, 27.96, 21.91, 27.94, 26.77, 21.79, 24.64, 27.25, 21.79, 24.89, 27.96, 25.00

Seq 3 12.88% 0.49	28.38, 21.90, 28.38, 23.20, 21.90, 28.38, 28.24, 21.90, 25.00, 28.38, 21.90, 28.38, 28.38, 22.24, 25.10, 28.38, 28.38, 24.72, 21.90, 28.37, 25.12, 27.13, 23.68, 21.90, 21.90, 28.38, 21.90, 24.35, 22.14, 28.24, 28.38, 23.78, 22.29, 28.38, 22.07, 25.35, 21.90, 28.38, 25.00, 21.90

Seq 4 12.60% 0.49	26.40, 27.75, 21.66, 27.90, 21.94, 27.91, 21.66, 22.67, 23.90, 27.64, 27.91, 21.88, 21.66, 21.66, 27.91, 27.91, 21.66, 27.03, 27.42, 26.29, 21.66, 25.65, 27.90, 21.66, 27.89, 25.77, 21.66, 21.66, 26.10, 27.91, 21.66, 24.73, 27.91, 21.66, 27.90, 24.01, 21.66, 27.90, 21.66, 23.63

**Table 2:** The errors between estimated and true solution at various AD rates for four sequences.

Seq. ID	SNR (dB)	AD rate (kHz)											
		Inf	1	2	3	4	5	7	9	11	15	20	25
Seq 1	9.5	2.02	20.53	12.65	10.31	8.85	7.87	6.67	5.94	5.29	4.58	3.96	3.54
	0	8.24	68.01	43.29	35.02	29.79	26.66	22.55	20.02	17.89	15.50	13.41	12.00
	−6.0	18.51	141.81	91.21	73.72	62.67	56.15	47.48	42.10	37.68	32.63	28.22	25.27

Seq 2	9.5	4.19	31.52	21.39	17.91	15.40	14.05	11.72	10.26	9.40	8.02	6.98	6.43
	0	9.52	78.32	52.98	44.67	38.33	35.15	29.23	25.55	23.44	19.99	17.44	6.23
	−6.0	18.14	149.64	101.20	85.47	73.32	67.31	55.94	48.87	44.83	38.25	33.39	31.15

Seq 3	9.5	5.02	27.32	18.70	15.48	13.67	11.78	10.07	8.83	8.00	6.85	5.96	5.32
	0	10.60	63.02	42.47	35.49	31.81	26.83	23.06	20.21	18.27	15.66	13.63	12.17
	−6.0	19.13	117.29	78.61	65.94	59.41	49.73	42.81	37.53	33.89	29.07	25.29	22.57

Seq 4	9.5	3.48	21.60	15.11	12.64	10.88	9.63	8.31	7.35	6.52	5.63	4.85	4.37
	0	10.79	67.96	47.93	39.82	34.17	30.32	26.14	23.13	20.61	17.75	15.30	13.76
	−6.0	22.53	139.85	98.80	81.91	70.28	62.39	53.76	47.56	42.44	36.51	31.48	28.31
